# Outcome of facial contour asymmetry after conventional two-dimensional versus computer-assisted three-dimensional planning in cleft orthognathic surgery

**DOI:** 10.1038/s41598-020-58682-4

**Published:** 2020-02-11

**Authors:** Po-Jung Hsu, Rafael Denadai, Betty C. J. Pai, Hsiu-Hsia Lin, Lun-Jou Lo

**Affiliations:** 1 Department of Surgery, Chang Gung Memorial Hospital, Taoyuan, Taiwan; 2 Department of Plastic and Reconstructive Surgery and Craniofacial Research Center, Chang Gung Memorial Hospital, Chang Gung University, Taoyuan, Taiwan; 3 Department of Craniofacial Orthodontics and Craniofacial Research Center, Chang Gung Memorial Hospital, Taoyuan, Taiwan; 4 Image Lab and Craniofacial Research Center, Chang Gung Memorial Hospital, Taoyuan, Taiwan

**Keywords:** Medical imaging, Medical imaging, Outcomes research, Outcomes research

## Abstract

Computer-assisted 3D planning has overcome the limitations of conventional 2D planning-guided orthognathic surgery (OGS), but difference for facial contour asymmetry outcome has not been verified to date. This comparative study assessed the facial contour asymmetry outcome of consecutive patients with unilateral cleft lip and palate who underwent 2D planning (n = 37)- or 3D simulation (n = 38)-guided OGS treatment for correction of maxillary hypoplasia and skeletal Class III malocclusion between 2010 and 2018. Normal age-, gender-, and ethnicity-matched individuals (n = 60) were enrolled for comparative analyses. 2D (n = 60, with 30 images for each group) and 3D (n = 43, with 18 and 25 images for 2D planning and 3D simulation groups, respectively) photogrammetric-based facial contour asymmetry-related measurements were collected from patients and normal individuals. The facial asymmetry was further verified by using subjective perception of a panel composed of 6 blinded raters. On average, the facial contour asymmetry was significantly (all p < 0.05) reduced after 3D virtual surgery planning for all tested parameters, with no significant differences between post-OGS 3D simulation-related values and normal individuals. No significant differences were observed for pre- and post-OGS values in conventional 2D planning-based treatment, with significant (all p < 0.05) differences for all normal individuals-related comparisons. This study suggests that 3D planning presents superior facial contour asymmetry outcome than 2D planning.

## Introduction

Orthognathic surgery (OGS) plays a key role for successful management of a myriad of facial deformities associated with malocclusion, with facial asymmetry feature being most frequently associated with skeletal class III malocclusion^1–3^. Particularly, patients with unilateral cleft lip and palate differ from the noncleft population, because they have a congenital orofacial defect compromising both the bone and soft tissues on the lesion side^4–6^, which may be expressed as facial asymmetry^7–15^. Skeletal mature patients with clefts frequently presents with maxillomandibular disharmony (maxillary hypoplasia and skeletal class III malocclusion) which requires OGS treatment^7–15^. As residual facial asymmetry after OGS treatment may negatively impact patients’ perceptions about outcome requiring further revisionary surgical interventions^16–19^, the significance of preoperative prediction of this deformity should not be underestimated^15,16,20^. Therefore, it is of paramount that an accurate diagnosis of asymmetry is accomplished preoperatively for that a precise surgical planning and execution is performed^7,15,16,20^.

In this setting, OGS planning has evolved over the past decades^15–24^. Traditionally, OGS planning has been constructed on two-dimensional (2D) cephalometry, 2D photographic analysis, articulators, and dental models^21,22^. However, this planning modality presents limitations for challenging clinical scenarios such as patients with clefts associated with malocclusion and asymmetry due to lack of prediction of rotational movements around the vertical axis (yaw rotation parameter) and also existence of distorted measurements^23^. On the other hand, three-dimensional (3D) surgical simulation can precisely detect the facial asymmetry and subsequently anticipate need-based surgical movements such as translational and rotational mobilization of the maxillomandibular complex in six different directions^15,16^.

A growing body of 3D technology-related literature has documented its accuracy and feasibility^16,24–28^. Comparative studies between 2D and 3D planning methods have also been published but with most of endpoints being established grounded on skeletal framework-based analysis^28–33^. However, the OGS-treated patients concerns are mainly centered on the facial soft tissue feature as it is the final response of bone movements and remodeling during postoperative follow up^15,17,19,20,27^. It is consequently fundamental that additional outcome studies are conducted by implementing a well-delineated methodology based on accurate quantitative and qualitative evaluation of facial soft tissue dimensions and comparisons between OGS-treated patients and normal individuals.

Historically, the facial asymmetry was mainly appraised using 2D radiograph or photography-guided landmark comparisons^21–23^. However, these tools may have underestimated the spatial 3D composition of the face, a complex structure with distinct anatomical components and contours^34–36^. Nowadays, 3D photogrammetry is considered an especially preferred tool for facial soft tissue analysis^34–36^. We are not aware of any comparative study of 2D and 3D planning modalities focused on soft tissue-based facial contour asymmetry outcome analysis using 3D photogrammetric tool.

The primary purpose of this study was to compare the objective and subjective treatment outcomes between conventional 2D and computer-assisted 3D planning by using 2D- and 3D facial soft tissue image-based facial contour asymmetry measurements in OGS-treated patients with unilateral cleft lip and palate. The secondary purpose was to compare the patients’ objective facial contour asymmetry values with the normal individuals-based facial contour asymmetry values.

## Results

This study included 75 OGS-treated patients with unilateral clefts in the 2D planning (n = 37) and 3D simulation (n = 38) groups. The groups have similar age at surgery, distribution for sex, and pattern of facial deformity (unilateral cleft lip and palate, maxillary hypoplasia, and class III malocclusion) (Table 1). They have specific patterns of distribution of images included for 2D- and 3D-based facial asymmetry analyses, with 30 (81.1%) and 30 (78.9%) patients presenting 2D photographic images with satisfactory quality in 2D planning and 3D simulation groups, respectively; and 18 (48.6%) and 25 (65.8%) patients presenting 3D facial images with satisfactory quality in the 2D planning and 3D simulation groups, respectively.

**Table 1:** Characteristics of Patients with Complete Unilateral Cleft Lip and Palate Included in this Study.

Parameters	2D planning group (*n* = 37)	3D simulation group (*n* = 38)
**Male/Female**, n (%)	21 (57) /16 (43)	19 (50) /19 (50)
**Age at surgery**, years (m ± sd)	18.6 ± 3.1	18.9 ± 2.7
**Genioplasty**, yes/no n (%)	16 (43) / 21 (57)	24 (63) / 14 (37)
**Postoperative follow up**, months (m ± sd)	16.7 ± 11.3	13.3 ± 5.6
**Available image for facial contour analysis**, n (%)		
2D full-face frontal view photographs	30 (81.1)	30 (78.9)
3D full-face photographs	18 (48.6)	25 (65.8)

n, number of subjects; m, mean; sd, standard deviation.

### 3D-based facial contour asymmetry analysis

**Figure 1:**
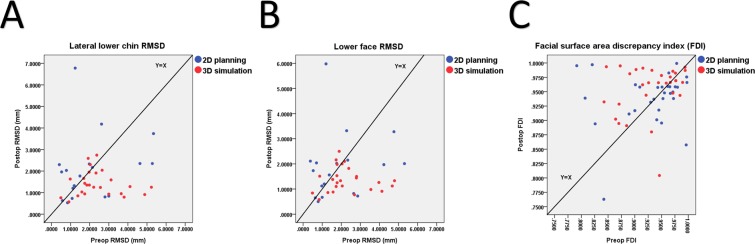
Scatter plots of pre- and post-orthognathic surgery data for root-mean-square-deviation (RMSD) and facial surface area discrepancy index (FDI) parameters for conventional two-dimensional planning and three-dimensional simulation groups.

For 2D planning group (n = 18), no significant differences were observed between pre- and post-OGS root-mean-square-deviation (RMSD) values for lateral lower chin or lower facial regions. For 3D simulation group (n = 25), the post-OGS RMSD values were significantly (p < 0.05) lower than pre-OGS RMSD values for lateral lower chin or lower facial regions (Tables 2 and 3; Fig. 1A,B).

**Table 2:** Quantitative Facial Contour Outcomes.

Parameters	2D planning group			3D simulation group			Normal group^§^ m ± sd (95% CI)
	Pre-OGS m ± sd (95% CI)	Post-OGS m ± sd (95% CI)	p	Pre-OGS m ± sd (95% CI)	Post-OGS m ± sd (95% CI)	p	
**RMSD, mm** ^*,**^							
Lower face	1.94 ± 1.51 (1.189–2.687)	1.88 ± 1.32 (1.225–2.536)	>0.05	2.29 ± 1.15 (1.818–2.766)	1.37 ± 0.49 (1.167–1.573)	**<0.001**	1.16 ± 0.39 (1.015–1.308)
Lateral lower chin	2.06 ± 1.60 (1.226–2.853)	2.11 ± 1.54 (1.344–2.879)	>0.05	2.41 ± 1.18 (1.919–2.896)	1.41 ± 0.58 (1.173–1.650)	**<0.001**	1.32 ± 0.42 (1.161–1.474)
FDI, % (m ± sd) ^†,‡^	0.93 ± 0.06 (0.909–0.952)	0.94 ± 0.05 (0.922–0.956)	>0.05	0.93 ± 0.05 (0.914–0.949)	0.95 ± 0.04 (0.938–0.971)	**<0.001**	0.97 ± 0.02 (0.964–0.978)
VAS, (m ± sd) ^†,‡^	5.48 ± 1.97 (5.188–5.767)	5.75 ± 1.66 (5.506–5.994)	>0.05	5.67 ± 1.91 (5.391–5.953)	6.33 ± 2.22 (6.001–6.654)	**<0.001**	–

Bold values indicate statistical significance after Bonferroni correction; m, mean; sd, standard deviation; CI, confidence interval; mm, millimeters; pre, preoperative; post, postoperative; OGS, orthognathic surgery; RMSD, root-mean-square-deviation; FDI, facial surface area discrepancy index; VAS, visual analogue scale; –, not applicable;

*from a total of 75 patients with clefts, 43 (57.3%) had 3D facial images, with 18 and 25 patients in 2D planning and 3D simulation groups, respectively;

**total of 86 3D images, with 18 pre-OGS and 18 post OGS images for 2D planning group and 25 pre-OGS and 25 post OGS images for 3D simulation group;

^†^from a total of 75 patients with clefts, 60 (80%) had 2D frontal photographic images, with 30 patients in each group;

^‡^total of 120 2D photographic images, with 30 pre-OGS and 30 post-OGS 2D photographic images for each group;

^§^from a total of 60 normal individuals, 30 (50%) had 3D facial images and other 30 (50%) had 2D frontal photographic images.

**Table 3:** Intergroup Comparisons for Quantitative Facial Contour Outcomes.

Parameters	Intergroup comparisons					
	p*	p**	p^†^	p^‡^	p^§^	p^¶^
**RMSD**						
Lower face	>0.05	>0.05	**<0.001**	**<0.001**	**<0.001**	>0.05
Lateral lower chin	>0.05	>0.05	**<0.001**	**<0.001**	**<0.001**	>0.05
FDI	>0.05	>0.05	**<0.001**	**<0.001**	**<0.001**	>0.05
VAS	>0.05	**<0.001**	—	—	—	—

Bold values indicate statistical significance after Bonferroni correction; For mean values refer to Table 2; Pre, preoperative; post, postoperative; OGS, orthognathic surgery; RMSD, root-mean-square-deviation; FDI, facial surface area discrepancy index; VAS, visual analogue scale; –, not applicable;

*pre-OGS 2D planning group versus pre-OGS 3D simulation group;

**post-OGS 2D planning group versus post-OGS 3D simulation group;

^†^pre-OGS 2D planning group versus normal group;

^‡^pre-OGS 3D simulation group versus normal group;

^§^post-OGS 2D planning group versus normal group;

^¶^post-OGS 3D simulation group versus normal group.

**Figure 2:**
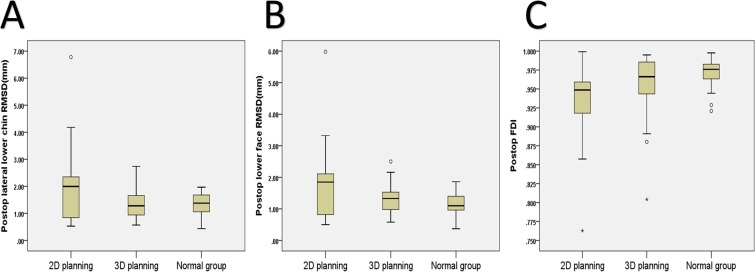
Box plots of post-orthognathic surgery data for root-mean-square-deviation (RMSD) and facial surface area discrepancy index (FDI) parameters for conventional two-dimensional planning and tree-dimensional simulation groups. The normal group data is also presented. The symbols “*” and “°” indicate the outliers.

Comparison between 3D simulation and 2D planning groups had no significant difference for pre and post-OGS RMSD values (Tables 2 and 3; Fig. 2A,B). The normal group (n = 30)-related RMSD values were significantly (p < 0.05) inferior than 2D planning group-related RMSD values for pre- and post-OGS periods. The normal group-related RMSD values were significantly (p < 0.05) inferior than 3D simulation group-related RMSD values for pre-OGS period, but with no significant difference for comparison between normal group and post-OGS 3D simulation group values (Tables 2 and 3; Fig. 2A,B).

The qualitative analysis revealed that a significant (p < 0.05) higher number of patients of 2D planning group presented RMSD values > 2 mm when compared with patients of 3D simulation group (Table 4).

**Table 4:** Comparisons for Qualitative Facial Contour Outcomes.

Parameters	2D planning group (*n* = 18)	3D simulation group (*n* = 25)	*p*
**Lower face**, ***n*** **(%)**			
Post-OGS RMSD < 2 mm	10 (56)	21 (84)	**<0.001**
Post-OGS RMSD ≥ 2 mm	8 (44)	4 (16)	
**Lateral lower chin**, ***n*** **(%)**			
Post-OGS RMSD < 2 mm	9 (50)	21 (84)	**<0.001**
Post-OGS RMSD ≥ 2 mm	9 (50)	4 (16)	

Bold values indicate statistical significance after Bonferroni correction; n, number of subjects; Post, postoperative; OGS, orthognathic surgery; RMSD, root-mean-square-deviation; mm, millimeters.

### 2D-based facial contour asymmetry analysis

For 2D planning group (n = 30), no significant differences were observed between pre- and post-OGS facial surface area discrepancy index (FDI) values. For 3D simulation group (n = 30), the average post-OGS FDI value was significantly (p < 0.05) superior to pre-OGS measurement (Tables 2 and 3; Fig. 1C).

Comparison between 3D simulation and 2D planning groups had no significant difference for pre and post-OGS FDI values (Table 3).

The normal group (n = 30)-related FDI values were significantly (p < 0.05) superior to 2D planning group-related FDI values for pre- and post-OGS periods. The normal group-related FDI values were significantly (p < 0.05) superior to 3D simulation group-related FDI values for pre-OGS period, but with no significant difference for comparison between normal group and post-OGS 3D simulation group values (Tables 2 and 3; Fig. 2C).

### Panel assessment

For 2D planning group (n = 30), no significant differences were observed between pre- and post-OGS visual analogue scale (VAS) scores. For 3D simulation group (n = 30), the average post-OGS VAS score was significantly (p < 0.05) superior to pre-OGS measurement (Table 2). Post-OGS VAS score-related 3D simulation group was significantly superior to post-OGS VAS score-related 2D planning group. Inter-observer reliability was considered high (Cronbach’s alpha = 0.973).

## Discussion

Previous comparative studies have shown that 3D simulation provides superior results for OGS treatment compared to 2D planning, with disparities between the studies for sample compositions, outcome measure tools, and endpoints of research^15,16,28–32,37–39^. This study was designed to investigate possible differences of facial contour asymmetry after OGS treatment in a cohort of patients with clefts, depending on either 2D- or 3D-guided planning modality. For this, we encompassed a detailed quantitative and qualitative evaluation of the asymmetry of contour in the lower third of the face from different perspectives, including the advanced biomedical engineering software-based 3D photogrammetry methodology, the traditional 2D photogrammetric analysis, and the 2D facial image-based panel assessment. Instead of using the widely applied landmarks-guided linear and angular measurements for determination of the facial asymmetry, the employed 2D- and 3D photogrammetric-based analyses take advantage of all facial surface-related data points which characterizes a more global and precise evaluation of the facial contour asymmetry. These photogrammetric facial surface-based methodologies were previously adopted by other groups testing different hypothesis, with the validity and reproducibility parameters being formerly demonstrated^39–42^. However, the adopted methods can be time-consuming and also be associated with limitations related to operator-dependent landmark and facial mid-sagittal plane identification. Importantly, the same blinded evaluator consistently performed all 2D- and 3D-guided photogrammetric measurements using unchanged landmarks and reference planes definitions; therefore, it was expected that intrinsic errors associated with the computerized-based systems would have been similar in all included patients and normal individuals, with no or minimum interference with the intragroup (pre- versus post-OGS) and intergroup comparisons.

For both 2D planning and 3D simulation groups, the average follow-up period was longer than 12 months, with the minimum time criteria for acquisition of facial photographic images to mitigate potential problems related to facial soft tissue swelling^43^. The involved patients had differences (mean of 3 months) for postoperative follow up parameter as it was related to the time required for orthodontic adjustment before debonding and it is broadly variable from patient to patient in different cohorts^44,45^. Our findings demonstrated that both groups had similar average pre-OGS facial contour asymmetry values for all photogrammetric- and panel-based tools, demonstrating that the interpretations of post-OGS comparative analyses may be performed with minimum influence from different degrees of preoperative deformity. Furthermore, we exhibited that even the included normal individuals presented with a certain degree of facial asymmetry as revealed by both the 2D and 3D photogrammetric methods. As this finding has widely been demonstrated for normal populations^34,46^, valid deductions could be made from normal-related comparisons.

Different from mid-face or nasolabial measurement-based outcome investigations^35,36,47^, in this study we addressed particularly the lower face region. In the literature, facial asymmetry is frequently observed in patients with skeletal Class III malocclusion, with the lower face representing the main anatomical site for presence of asymmetries compared to upper and middle face regions^1–3^. In recent studies addressing specifically clefts, cone beam computed tomography (CBCT) image-based analysis have demonstrated that patients with unilateral cleft lip and palate show a more severe lower face asymmetry than patients with bilateral cleft lip and palate and non-cleft patients with similar class III skeletal relationships^12–14^. In our study, the normal individuals had significantly lower RMSD and higher FDI values than OGS-treated patients in the pre-OGS period, regardless of tested group. Therefore, in addition to the typical cleft-associated nasolabial asymmetric deformity^35,36,47^, the lower third of the face appears^12–14^ to be an additional leading factor in facial asymmetry in patients with unilateral cleft lip and palate.

Meticulous treatment of patients with combined abnormalities (such as clefts, malocclusion, and asymmetry) may require a precise mobilization of the maxillary and mandibular segments to address all possible deformities in a single procedure^15,16,26^. Based on the evolving experience of our center^15,16,26,30,45^, the single-splint two-jaw OGS approach provides enormous surgical flexibility for the treating surgeon, allowing the positioning of the maxillomandibular complex in six degrees of free movement with achievement of the desired position that yields the best balance between functional occlusion and facial esthetic and symmetric results. However, this multidirectional movements of maxillomandibular complex cannot be precisely anticipated by using the conventional 2D planning^21,22^. Particularly, the yaw rotation of maxillomandibular complex and the positioning of proximal mandible ramus segments, two key features for facial asymmetry correction^15,16,26^, may not be predicted by 2D planning^21,22^.

Employing the 3D simulation for OGS planning, the orthodontic and surgical professionals may interactively judge the skeletal framework changes after final surgical occlusion setup and also contemplate the bone framework morphology and its relationship to the soft tissue envelope. The complete judgment of frontal, profile, and basal views allows that the translational and rotational movements of the maxilla and the proximal and distal segments of the mandibular ramus are accurately tailored to need of each patient under treatment^15,16,26^. Using this detailed 3D planning, we demonstrated that patients with cleft-associated deformity (maxillary hypoplasia, class III malocclusion, and asymmetry) had improvement of facial contour asymmetry after OGS treatment, with similar post-OGS facial contour asymmetry values to normal individuals for all tested 2D and 3D photogrammetric-based objective methods. The 2D planning group composed by patients with similar deformities did not have significant changes in facial contour asymmetry after OGS treatment, as previously demonstrated^17,48^. Furthermore, the 2D planning group had inferior post-OGS facial contour asymmetry outcome than normal individuals for all tested photogrammetric methods.

Considering only the post-OGS quantitative RMSD and FDI tools-based comparative analyses, one may imply that 2D planning and 3D simulation modalities resulted in a similar facial contour asymmetry outcome. However, a detailed scrutiny of our quantitative results reveals further interesting findings. The overall average values to assess the differences between 2D planning and 3D simulation groups may level out the asymmetry scores, because lower asymmetry values are levelled out by higher ones to create the mean as revealed by the Figs. 1 and 2. The consideration of box plots in Fig. 2 demonstrates that the 3D simulation provided a more consistent facial contour asymmetry outcome (Supplementary Fig. S1), with the 3D simulation group exhibiting a small difference between the upper and lower quartiles compared to 2D planning group. Furthermore, although with no significant difference, the average post-OGS RMSD-based lateral lower chin value was higher than the pre-OGS value in 2D planning group, suggesting that this particular region was not completely addressed during preoperative planning and surgical execution. The Fig. 1 also displays that some of post-OGS RMSD values were higher than pre-OGS RMSD values mainly in the 2D planning group, indicating that the facial contour asymmetry was even aggravated postsurgery for the two measured regions (Supplementary Fig. S2). Similar findings were observed for FDI values. This worsening of asymmetry was probably secondary to the lack of prediction of chin region and proximal ramus mandibular changes including the rotational movements and the intersegmental bone gap differences between the right and left sides of the face. We hypothesize that studies embracing a large sample size may demonstrate significant differences for the average values of these quantitative measure outcome tools.

In addition to these quantitative photogrammetric parameters, we qualitatively stratified the patients using the threshold value of 2 mm for RMSD tool. It revealed that a higher number of 2D planning-guided OGS-treated patients had RMSD values above the threshold value that could be detected in clinical evaluation. A combination of patients’ desires and professionals’ judgements have been considered important in decision-making process for surgical correction of asymmetries considered “borderline” with values close to the discriminative threshold^7,34^. However, in severe asymmetries (most perceptible), the decision for treatment is usually simple with higher indication for surgical management^1–3,15,16,18,30^. Our RMSD threshold-related finding would allow OGS professionals to counsel future patients based on objective 3D photogrammetric-based data.

We also verified the subjective perceptions from blinded raters using the panel assessment, an outcome measure tool frequently embraced in OGS literature^49^. Interestingly, the post-OGS panel assessment-based results revealed that raters appraised the patients treated with 3D simulation as more symmetrical than 2D planning-treated patients, proposing that the facial changes after OGS treatment impacted the raters’ perceptions. The subjective results from lay raters are clinically plausible as it acts as a simulated environment of social interactions in daily life and also symbolizes the external validity in terms of the public members’ observations of facial deformity and its surgical management^50,51^.

The absence of a study with similar methodological design impair any attempt for a truthful head-to-head comparison between the existing findings and our current results. We assessed facial contour asymmetry because it is a relevant clinical repercussion of facial bone mobilization and remodeling and the end-point of OGS treatment for the management of contour deformities. Other groups assessing cost-effectiveness and patient-reported outcomes endpoints have also demonstrated favorable results for 3D planning modality^15,16,23–27^. Therefore, based on the current findings alongside with the previous results^15,16,23–27^, it can be indicated that the 3D simulation should be the planning method of choice for achievement of lower face contour symmetry in patients with unilateral cleft, maxillary hypoplasia, and class III malocclusion.

**Figure 3:**
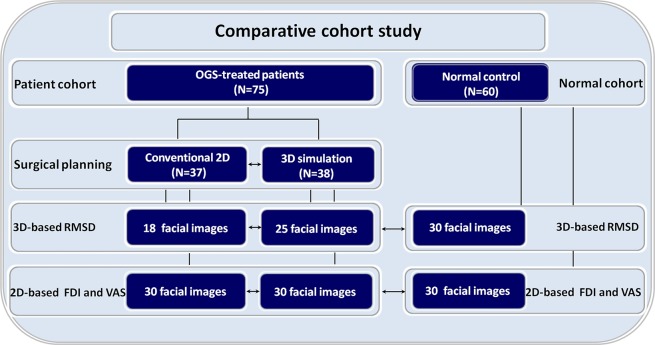
Flowchart for study design with three-dimensional (3D) and two-dimensional (2D) and facial images-based data collection (root-mean-square-deviation, RMSD; facial surface area discrepancy index, FDI; and visual analogue scale, VAS) for orthognathic surgery (OGS)-treated patients with unilateral cleft lip and palate (conventional 2D planning and 3D simulation groups) and normal individuals. Single arrows indicate comparison analysis.

Potential caveats of this study should be addressed. Some of included patients did not had both 3D and 2D imaging records matched by postoperative time, generating the sample differences for each of tested parameters (Table 2; Fig. 3). Our findings are restricted to a specific subgroup of young adult patients with unilateral clefts who were managed by a particular OGS technique (single splint two-jaw OGS procedure with 3D printed surgical wafer but with no 3D printed surgical guides), and any generalizations must be undertaken with caution. The adoption of a standardized approach as previously described by our team^15,16,26^ partially limited the bias of orthodontic and surgical technique- and professional-related factors while interpreting our comparative results, but differences between the embraced patients were expected for types of the maxillary and mandible movements and presence of genioplasty as it was defined according to the planning modality. Future investigation may address the impact of specific types of bone movements and other facial regions (e.g., paranasal and midface areas) when comparing the 2D and 3D-based OGS planning outcomes. A further study should also be conducted to compare different types of transfer of 3D planning to actual surgery (e.g., with and without 3D printed surgical guides) as well as different surgical approaches (e.g., single-jaw and two-splint two-jaw procedures) in terms of facial contour asymmetry outcomes.

In conclusion, this OGS outcome-based study suggests that 3D simulation presents superior facial contour asymmetry outcome to 2D planning.

## Methods

### Study population

This comparative retrospective cohort study (Fig. 3) was conducted on consecutive skeletally mature patients with complete unilateral cleft lip and palate who had undergone 3D image-guided OGS treatment (3D simulation group) for correction of maxillary hypoplasia and skeletal Class III malocclusion by the senior orthodontic and surgical professionals (BCJP and LJL) at Chang Gung Craniofacial Center between August 2014 and January 2018. Consecutive patients with similar diagnosis who had undergone 2D planning-guided OGS treatment (2D planning group) for analogous indications between January 2010 and July 2014 by the same professionals were included for comparative analysis. All included patients had undergone initial surgeries (lip, palate, and alveolar repairs) during the growing age according to the previously published institutional protocol^52^. Demographic, clinical, and outcome data were collected. Patients with any associated syndromes; those who had undergone further bone or soft tissue surgical intervention (e.g., bone contouring, rhinoplasty, lip revision, or fat grafting) within the OGS procedure and image acquisition period; and those with no adequate 2D or 3D imaging or no complete follow-up observation (<6 months after OGS procedure) were excluded.

2D and 3D facial images from 60 (30 sets of 2D data and 30 sets of 3D data) normal Taiwanese Chinese individuals (no obvious facial asymmetry and no history of craniofacial deformity, syndrome, trauma, or surgery; normal group) were retrieved from the Chang Gung Craniofacial Research Center database, adjusted for matching factors (age and sex), and used for comparative analysis.

### Ethics

This retrospective study was conducted and approved by Chang Gung Craniofacial Center, Taiwan. All experiments were performed with the approval of the Institutional Review Board (IRB) of Chang Gung Memorial Hospital (IRB 20170033B0) and the study methods were carried out in accordance with the approved guidelines of IRB. Informed consent from guardians was obtained for those patients who are below 18 years of age. All patients of 18 years of age or older provided their own informed consent for participation. Informed consent for publication of identifying information/images in an online open-access publication was obtained from the patient displayed in this article.

### Orthognathic surgery treatment

Except for the difference in preoperative planning modality, both included groups were treated equally during study period. All the involved patients received modified surgery first model and single-splint two-jaw OGS with or without genioplasty according to the previously described surgical approach principles^15,16,26^. The patients with no intermaxillary fixation were admitted in regular ward for 2 days following the surgery and then clinically examined based on regular surgical and orthodontic appointments. A liquid diet was advised in the first week, followed by a soft diet in the second week. Orthodontic treatment was performed for arch form compatibility (leveling, alignment, arch coordination, and dental decompensation) before surgery and continued after surgery.

### 2D planning

For the 2D planning group, preoperative 2D cephalogram, 2D photographs, and dental casts were adopted for conventional 2D surgical planning based on the cephalometric analysis and normal Taiwanese Chinese cephalometric data. For the paper-surgery planning, the lateral and frontal cephalograms were traced with acetate matte paper; the maxilla and mandible templates were then cut out from both cephalogram tracings in order to simulate the desired movements. For the model-surgery planning, the dental casts were mounted in an adjustable articulator with face bow transfer and wax checkbite registration. The 2D planning-guided model surgery (maxilla and mandible positioning) was performed, and the final occlusal splint was fabricated manually. OGS procedure was then executed using the 2D surgical planning and the final occlusal splint as guides. No planning software was used in this group.

### 3D planning

For the 3D simulation group, all technical features were previously detailed by our group^15,16,26^. In addition to conventional 2D surgical planning, all patients of this group had preoperative CBCT image scans obtained using an i-CAT scanner (Imaging Sciences International, Hatfield, PA, USA) with the following parameters: 120 kVp, 0.4 × 0.4 × 0.4-mm voxel size, 40-second scan time, and 22 × 16-cm field of view. Dental casts were digitalized by using surface scanner (3Shape, Copenhagen, Denmark).

**Figure 4:**
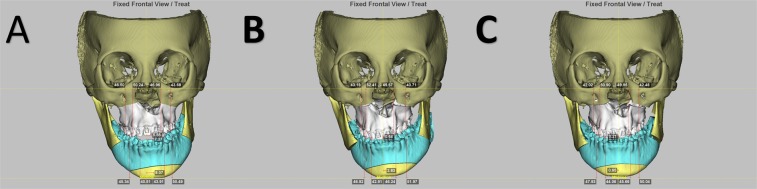
Example of three-dimensional simulation for single-splint two-jaw orthognathic surgery in a patient with left cleft and obvious facial asymmetry. (**A**) Using the preoperative cone beam computed tomography-based image, the maxillo-mandibular complex was created by segmenting the maxilla and mandible. (**B**) The planning was transferred into three-dimensional model by mobilization of mandible segment using the occlusion set up as guide. (**C**) Modifications were implemented by mobilization of maxillo-mandibular complex and proximal ramus segment in translational and rotational directions for achievement of a balanced skeletal harmony and symmetry.

Using Dolphin 3D software package (Dolphin Imaging & Management Solutions, Chatsworth, California, USA) for CBCT image-based simulation, the maxillo-mandibular complex was created by segmenting the maxilla and mandible. The dentition in CBCT was replaced by the digitalized dental image to optimize the occlusal relationship (Fig. 4A). The conventional 2D surgical planning was then transferred into 3D model (Fig. 4B). For this, the maxillo-mandibular complex was mobilized as a single unit to match the surgical planning. To achieve skeletal harmony and facial symmetry, the maxillo-mandibular complex was further mobilized in feasible directions including translation as well as roll, pitch, and yaw rotation movements using the frontal, profile, and basal views, respectively (Fig. 4C). Potential bony collisions in the pterygomaxillary junction and mandible ramus regions were also carefully checked and modifications implemented accordingly. Genioplasty was finally simulated according to individual patient’s necessity. The fabrication of computer-generated 3D surgical wafers was accomplished for the 3D simulation group by adopting only the final surgical occlusion set up as template. For this, the digitalized dental image was initially manipulated by using the Dolphin 3D software for achievement of desired final surgical occlusion. This image data set was further adjusted (thickness) in the OrthoAnalyzer software package (3Shape, Copenhagen, Denmark) and then printed (Objet30 OrthoDesk 3D Printer, Stratasys Ltd., Israel) using a biocompatible PolyJet photopolymer material (MED610; Stratasys Ltd., Israel).

OGS procedure was then executed using the 3D printed final surgical wafer and 3D simulated image-related numerical and visual information (bone positioning and spatial relationship between osteotomized bone segments) as guiding templates for positioning of maxillo-mandibular complex and proximal mandible ramus and rigid fixation. In addition to the 3D printed surgical wafer, bone-guided measurements (medial and lateral maxillary pillars bilaterally), modified face bow-based midline checking (nasal dorsum and tip, lips, maxilla, dental arches, and chin points), and occlusal plane and middle and lower facial third proportions judgments were used for transferring the 3D planning to actual surgery. No 3D printed custom-made surgical guides or fixation plates were adopted in this group.

### Image acquisition for outcome analysis

For appraisal of facial contour asymmetry outcomes, two varieties of facial soft tissue images were employed in patients and normal individuals. Pre- and post-OGS standardized 3D facial images acquired using the 3dMD system (3dMD LLC, Atlanta, GA, USA) under standard conditions (a permanent installation with fixed ambient lighting and system and fixed individual positioning, including individuals with a natural head position, relaxed facial positioning, a closed mouth, and a thin elastic nylon cap to keep the hair away from the face)^34,35^ were used for objective assessment of 3D-based facial contour asymmetry analysis with quantitative and qualitative appraisal of data.

Pre- and post-OGS standardized 2D full-face frontal view photographs^53^ were used for 2D-based facial contour asymmetry analysis and panel assessment (objective and subjective methods, respectively) with quantitative appraisal of data.

### 3D-based facial contour asymmetry analysis

Pre (n = 43)- and post (n = 43)-OGS 3D images were adopted for facial contour asymmetry analysis, with 18 and 25 sets of pre- and post-operative images for 2D planning and 3D simulation groups, respectively. The degree of 3D facial contour asymmetry was obtained by calculating the quantitative root-mean-square-deviation (RMSD) value between the left and right sides of face, with higher RMSD values indicating a more asymmetric face. Reference planes and measurements were standardized based on previous 3D facial photogrammetric studies^54–57^. All pre- and post-OGS measurements were performed using Geomagic studio software package (3D system, Rock Hill, SC, USA).

**Figure 5:**
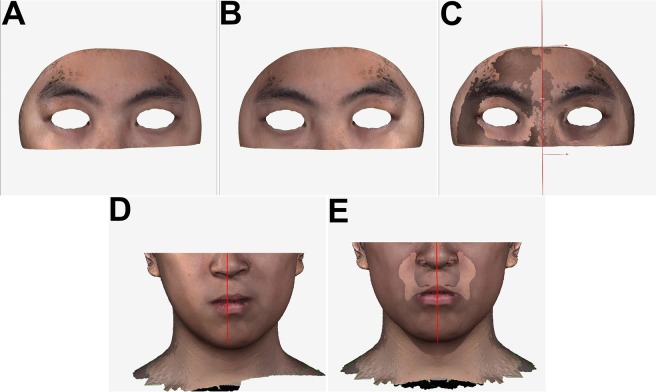
Determining the mid-sagittal plane for evaluation of the three dimensional-based facial contour asymmetry. (**A**) Removing the hair, ears, eyes, and infra-orbital rim regions from the original face of the patient before the orthognathic surgery treatment. (**B**) Flipping the original upper face to obtain a mirror upper face image. (**C**) Superimposition of the facial images displayed in A and B items by using the best-fit algorithm method. (**D**) Computer-guided automatic definition of the optimal mid-sagittal plane. (**E**) Simultaneous showing of the original and mirror full faces according to the automated computer-generated mid-sagittal plane.

**Figure 6:**
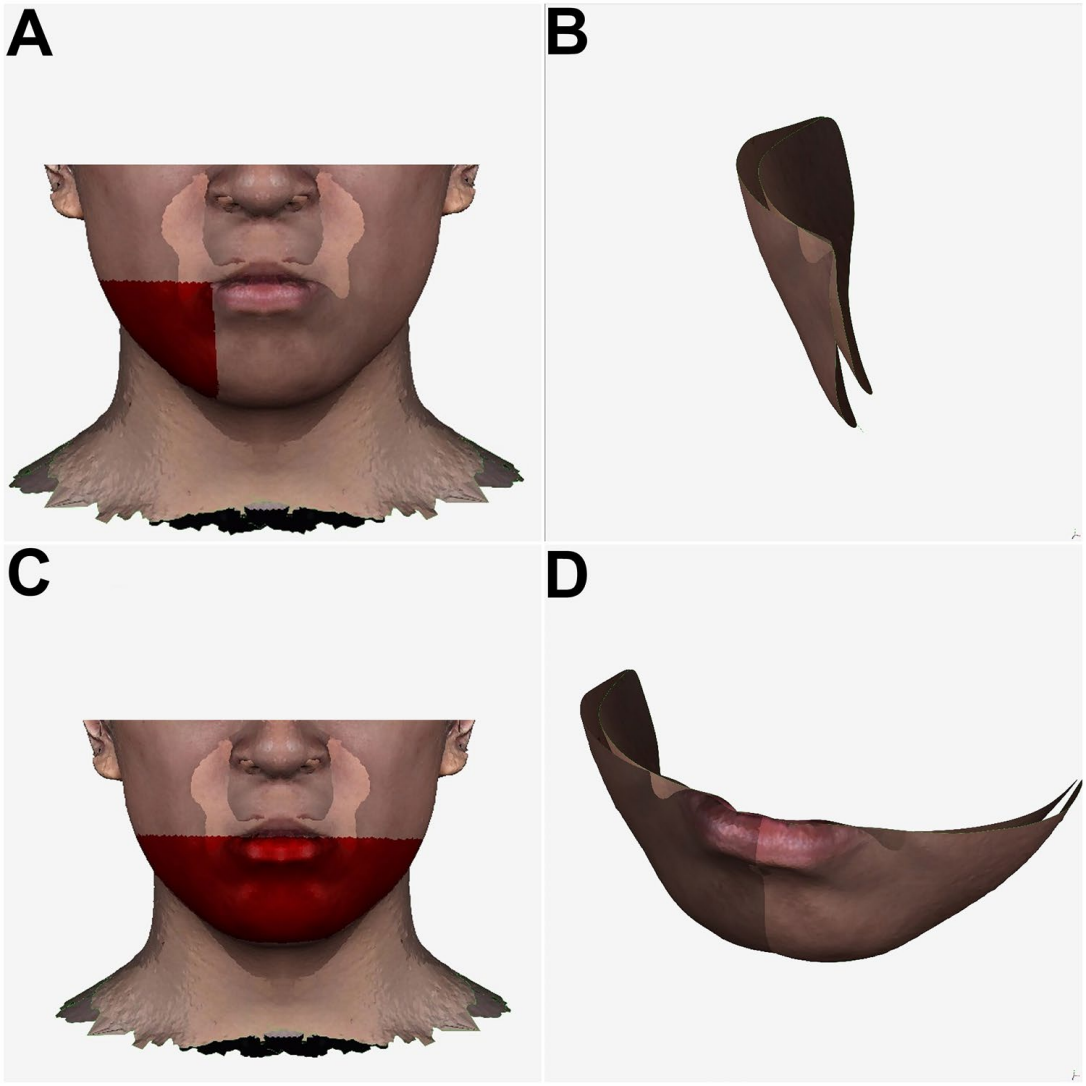
(**A**) Selection and (**B**) calculation of the contour asymmetry of lateral lower chin area by the root-mean-square deviation tool between the original and mirror faces. (**C**) Selection and (**D**) calculation of the contour asymmetry of lower face area by the root-mean-square deviation tool between the original and mirror faces.

To establish the facial mid-sagittal plane, the upper facial area (non-affected by OGS procedure) above the infra-orbital rim was selected and the hair, ears, and eyes areas were removed (Fig. 5A,B). A mirror upper face was created by flipping the original upper face with a temporarily mid-sagittal plane (Fig. 5B). The original and mirror upper faces were superimposed by using best-fit algorithm method (Fig. 5C), automatically generating an optimal mid-sagittal plane. This automated computer-generated mid-sagittal plane was applied to original whole face (Fig. 5D). The mirror whole face was then obtained by flipping the original whole face (Fig. 5E). The RMSD value was calculated between the original and mirror faces for two regions of interest: the lower face and lateral lower chin regions (Fig. 6).

To assess the post-OGS qualitative facial contour asymmetry outcome, all patients were stratified using the threshold of 2 mm deviation for RMSD values for each of assessed facial region^58–62^.

### 2D-based facial contour asymmetry analysis

**Figure 7:**
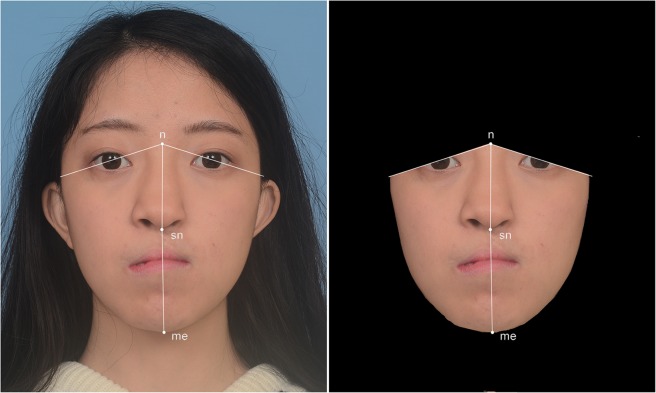
The two-dimensional photogrammetry-based facial surface area discrepancy index (FDI) method. n, nasion; sn, subnasale; me, menton. Informed consent for medical photographs was obtained from the patient.

Pre (n = 60)- and post (n = 60)-OGS 2D frontal photographic view images were adopted for facial contour asymmetry analyses, with 30 sets of pre- and post-operative images for both 2D planning and 3D simulation groups. A previously described facial surface area discrepancy index (FDI) method was employed for computerized photogrammetric facial contour asymmetry analysis^63–68^. Facial midline landmarks, including the nasion, subnasale, and menton, were marked. A vertical axis was drawn from these facial midline landmarks to divide the face in half. The upper boundary of the face was defined by a horizontal bipupillary line. The boundary of cheek and chin regions were manually defined, and the surface area of each hemiface was then automatically measured by the software (Fig. 7; Informed consent for medical photographs was obtained from the patient displayed in this figure). All pre- and post-OGS measurements were performed using Adobe Photoshop CS6 software package (Adobe systems, San Jose, CA, USA). Quantitative FDI scores were calculated by a previously adopted mathematical formulae^63^: FDI = (smaller surface area/larger surface area) x 100.

### Panel assessment

A panel composed of six blinded raters (no relationship to the included patients and not aware of the purpose of study) with no specialized professional training (i.e., no dental or medical background) was used for subjective assessments of the lower face asymmetry. The pre (n = 60)- and post (n = 60)-OGS 2D frontal photographic view images (30 sets of pre- and post-operative images for both 2D planning and 3D simulation groups) were presented in a fixed random sequence in a timed Microsoft PowerPoint presentation (Microsoft Corporation, Redmond, WA, USA) on a 15-inch MacBook Pro (Apple, Inc., Cupertino, CA, USA). All images were rated using a previously published qualitative rating system^63^: visual analogue scale (VAS) ranging from 1 to 10 (the most asymmetry and symmetry of the lower face region, respectively). The average scores between the raters were adopted for analysis.

### Statistical analysis

Descriptive statistical data are summarized as mean ± standard deviation and 95% confidence intervals. The data distribution was verified using the Kolmogorov–Smirnov test, and the paired *t*-test, two sample *t*-test, chi-square test, Kruskal–Wallis test, and Wilcoxon signed-rank test were performed accordingly. A Bonferroni correction was applied for multiple comparisons. Cronbach’s alpha reliability coefficient was used for inter-observer reliability of panel assessment. Two-sided p values < 0.05 were considered statistically significant. All analyses were performed using IBM SPSS software version 22.0 (IBM Corp., Armonk, NY, USA).

## Supplementary information

Supplementary information.
