# Supplementary material for: Outcome of facial contour asymmetry after conventional two-dimensional versus computer-assisted three-dimensional planning in cleft orthognathic surgery
Source: Sci Rep. 2020 Feb 11;10:2346. doi: 10.1038/s41598-020-58682-4 (PMC7012815; doi:10.1038/s41598-020-58682-4)
Supplement: Supplementary file 1 — Supplementary information. [file 41598_2020_58682_MOESM1_ESM.pdf]

Outcome of facial contour asymmetry after conventional two-dimensional versus computer-assisted three-dimensional planning in cleft orthognathic surgery

Po-Jung Hsu, Rafael Denadai, Betty CJ Pai, Hsiu-Hsia Lin & Lun-Jou Lo

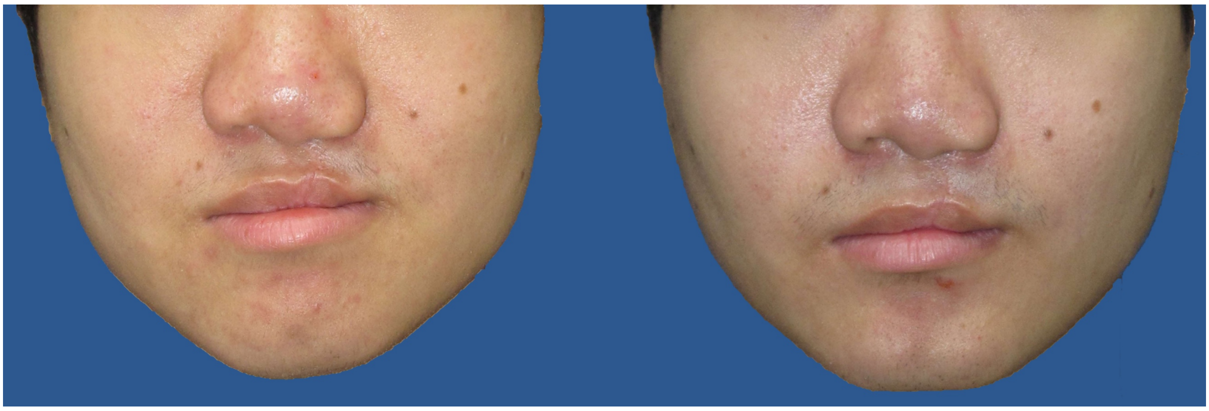

Supplementary Fig. S1. This is a 19 years old male with complete left cleft lip and palate, Class III malocclusion and face with hypoplastic appearance and asymmetry before (left) and 1 year after (right) three-dimensional simulation-guided orthognathic surgery who was satisfied with the facial contour symmetry and functional occlusion postsurgery.

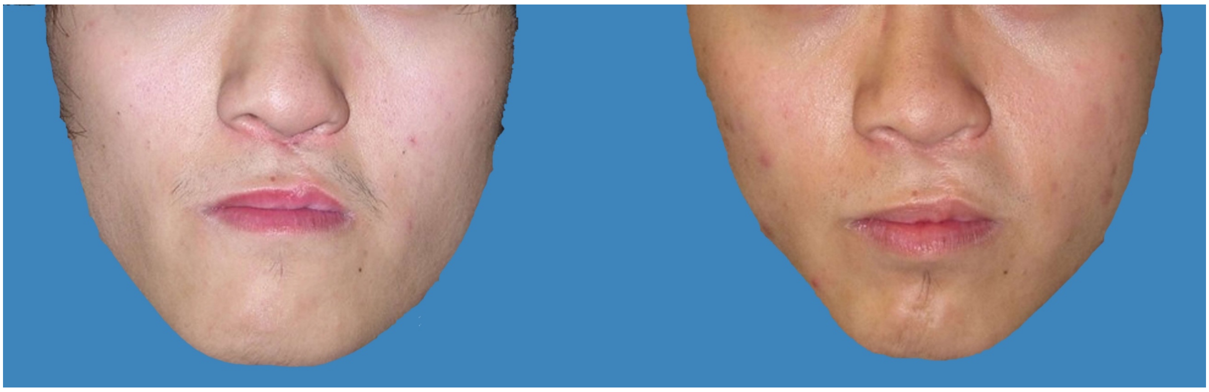

Supplementary Fig. S2. This is a 19 years old male with complete left cleft lip and palate, Class III malocclusion, hypoplastic appearance and asymmetry before (left) and 1 year (right) after two-dimensional planning-guided orthognathic surgery who was not satisfied with the facial contour asymmetry postsurgery, despite successful functional occlusion correction.
